# Current advance on distal myopathy genetics

**DOI:** 10.1097/WCO.0000000000001299

**Published:** 2024-07-16

**Authors:** Johanna Ranta-aho, Mridul Johari, Bjarne Udd

**Affiliations:** a Folkhälsan Research Center; b Department of Medical Genetics, Medicum, University of Helsinki, Helsinki, Finland; c Harry Perkins Institute of Medical Research, Centre for Medical Research, University of Western Australia, Nedlands, Western Australia, Australia; d Tampere Neuromuscular Center, Tampere University and Tampere University Hospital, Tampere, Finland

**Keywords:** distal myopathy, multisystem proteinopathy, oculopharyngodistal myopathy

## Abstract

**Purpose of review:**

Distal myopathies are a clinically heterogenous group of rare, genetic muscle diseases, that present with weakness in hands and/or feet at onset. Some of these diseases remain accentuated in the distal muscles whereas others may later progress to the proximal muscles. In this review, the latest findings related to genetic and clinical features of distal myopathies are summarized.

**Recent findings:**

Variants in *SMPX*, *DNAJB2,* and *HSPB6* have been identified as a novel cause of late-onset distal myopathy and neuromyopathy. In oculopharyngodistal myopathies, repeat expansions were identified in two novel disease-causing genes, *RILPL1* and *ABCD3.* In multisystem proteinopathies, variants in *HNRNPA1* and *TARDBP*, genes previously associated with amyotrophic lateral sclerosis, have been shown to cause late-onset distal myopathy without ALS. In *ACTN2*-related distal myopathy, the first recessive forms of the disease have been described, adding it to the growing list of genes were both dominant and recessive forms of myopathy are present.

**Summary:**

The identification of novel distal myopathy genes and pathogenic variants contribute to our ability to provide a final molecular diagnosis to a larger number of patients and increase our overall understanding of distal myopathy genetics and pathology.

## INTRODUCTION

Distal myopathies are rare, genetic, primary muscle disorders characterized by initial muscle weakness in the distal extremities. The affected areas are subjected to progressive atrophy and may include muscles of the forearms, hands, lower legs, and feet [1,2]. It is a highly heterogenous group of diseases with varying age of onset, inheritance pattern, clinical presentation, disease progression, and histopathological findings [1]. Disease-causing genetic variants have been identified in over 30 different genes (Table 1) [1,3]. The widespread application of high-throughput sequencing methods has accelerated the discovery of these genes in the last 10–15 years [1]. These methods have allowed us to sequence patients in increasing numbers using improved methods, and thus, generate vast amounts of sequencing data, shifting the bottle neck of molecular diagnosis from sequencing methods to data analysis and variant interpretation. More advanced sequencing methods, such as long read sequencing and optical genome mapping, have improved our ability to detect elusive variants [4]. Despite these advancements, many distal myopathy patients still remain without a molecular diagnosis. This is partly due to elusive causative variants and the observed clinical heterogeneity that makes the interpretation of identified variants more complicated. In addition, a fraction of these patients remains undiagnosed probably because they harbor variants in genes that have not yet been associated with skeletal muscle disease or within the ‘dark genome’, where noncoding regions have poorly understood functions that may impact gene expression and regulation. This review highlights the most recently identified genes that cause distal myopathy and other recent developments in distal myopathy genetics. 

**Box 1:**
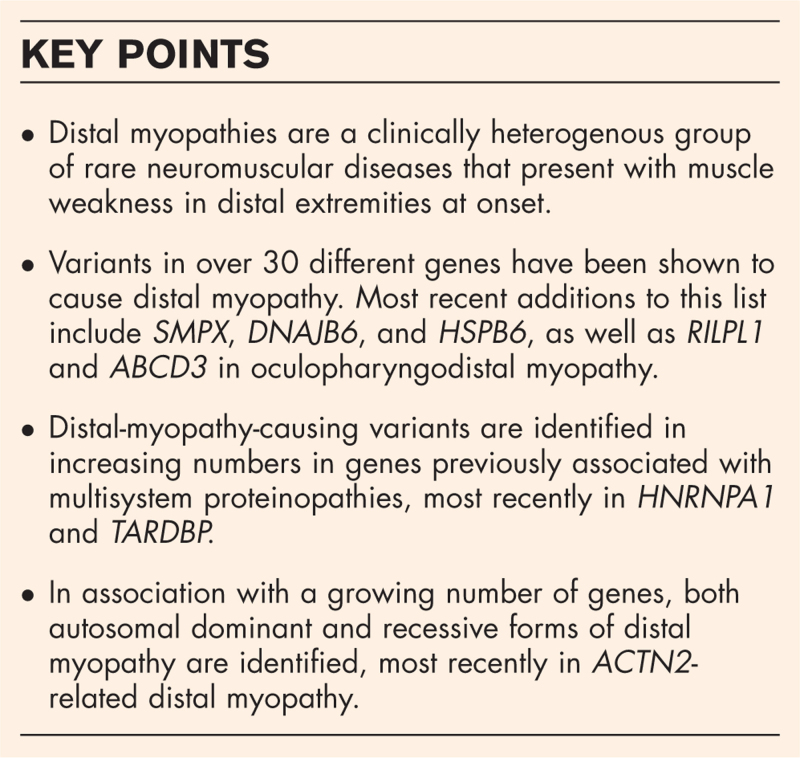
no caption available

**Table 1:** Distal myopathies by genetic definition

Gene	Protein	Protein function	Inheritance	Early weakness	Pathology
*ACTN2*	Alpha-actinin-2	Actin filament crosslinking	AD/AR	Anterior lower leg	Rimmed vacuoles
*ADSSL1*	Adenylosuccinate synthase-like 1	Catalyzation of IMP to AMP	AR	Posterior lower leg, calf	Myofibrillar, nemaline rods
*ANO5*	Anoctamine-5	Calcium-activated chloride channel, membrane repair	AR	Posterior lower leg, calf	Myopathic
*CAV3*	Caveolin-3	Component of caveolae, sarcolemma stabilization	AD	Hands and feet	Myopathic
*CRYAB*	Alpha-B crystallin	Chaperone-like activity, prevents aggregation of various proteins under stress conditions	AD	Anterior lower leg	Myopathic
*DES*	Desmin	Muscle specific intermediate filament protein family member, structure of the sarcomere	AD/AR	Anterior lower leg, scapula	Dystrophic, rimmed vacuoles, myofibrillar
*DNAJB2*	DNAJ subfamily B member 2	Prevention of irreversible protein aggregation	AD	Posterior lower leg, calf	Myofibrillar
*DNAJB6*	DNAJ subfamily B member 6	Prevention of irreversible protein aggregation	AD	Posterior lower leg, calf	Myofibrillar
*DYSF*	Dysferlin	Membrane repair	AR	Posterior lower leg, calf	Dystrophic
*FLNC*	Filamin C	Sarcomere structural integrity	AD	Hands	Myopathic
*GNE*	UDP-N-acetylglucosamine 2-epimerase	Sialic acid synthesis, rate limiting enzyme siaylation of muscle gylcans	AR	Anterior lower leg	Rimmed vacuoles
*LDB3*	Lim domain-binding 3	Sarcomere structural integrity	AD	Anterior lower leg	Desmin myotilin aggregates, rimmed and nonrimmed vacuoles
*HSBP6*	Small heat shock protein 22-kD protein 6	Z-disk maintenance, autophagy	AD	Anterior lower leg	Dystrophic, rimmed vacuoles and eosinophilic inclusions
*HSBP8*	Small heat shock protein 22-kD protein 8	Z-disk maintenance, autophagy	AD	Anterior lower leg	Dystrophic, rimmed vacuoles and eosinophilic inclusions
*MYH7*	Slow beta myosin heavy chain	Chemical energy into mechanical force	AD/AR	Anterior lower leg	Hypotrophy of type 1 slow muscle fibers
*MYOT*	Myotilin	Thin filament stabilization, Z-disk organization	AD	Posterior lower leg	Myofibrillar
*NEB*	Nebulin	Regulation of actin–myosin interaction, actin filament maintenance	AD/AR	Anterior lower leg	Myopathic with or without nemaline rods
*PLIN4*	Perilipin-4	Coat protein involved in biogenesis of lipid droplets	AD	Anterior lower leg	Myopathic, rimmed vacuoles
*RYR1*	Ryanodyne receptor 1	Calcium release in sarcoplasmic reticulum	AD/AR	Myalgia in calf	Cores
*SMPX*	Small muscle protein X-linked	Protection of the sarcolemma plasma membrane from mechanical stress	AD	Hands, finger extensors, anterior lower leg	Myopathic, rimmed vacuoles
*TTN*	Titin	Structure and flexibility of the sarcomere	AD/AR	Anterior lower leg, TA	Dystrophic rimmed vacuoles
Oculopharnygodistal myopathies					
*ABCD3*	ATP-binding cassette subfamily D member 3	Transport of branched fatty and bile acids into the peroxisome	AD	Ptosis	Rimmed vacuoles
*GIPC1*	GIPC PDZ domain-containing family member 1	Scaffolding protein, regulates cell surface receptor expression and trafficking	AD	Lower leg	Rimmed vacuoles
*LRP12*	Low-density lipoprotein receptor-related protein 12	LDL receptor-related protein, may function in signal transduction and/or endocytosis	AD	Lower leg	Rimmed vacuoles
*NOTCH2NLC*	NOTCH2 N-terminal-like C	Notch signaling and promotion of cortical neurogenesis	AD	Lower leg	Rimmed vacuoles
*RILPL1*	Rab-interacting lysosomal protein-like 1	Cell shape and polarity, protein transport, sequestering of GAPDH	AD	Ptosis	Rimmed vacuoles
Multisystem proteinopathies					
*HNRNPA1*	Heterogenous nuclear ribonucleoprotein A1	Regulation of protein expression through alternative splicing	AD	Hands	Rimmed vacuoles
*MATR3*	Matrin 3	Transcription, RNA splicing, DNA replication	AD	Anterior lower leg	Rimmed vacuoles
*TARDBP*	TAR DNA-binding protein	Nuclear RNA/DNA-binding protein, RNA processing, and metabolism	AD	Anterior and lateral lower leg, hands	Rimmed vacuoles
*TIA1+SQSTM1*	TIA1;Sequestosome-1	Splicing regulation and translation repression; ubiquitin-mediated autophagy;	DG	Hands, finger extensors	Rimmed vacuoles
*TIA1*	TIA1	Splicing regulation and translation repression	AD/AR	Hands, finger extensors	Rimmed vacuoles
*VCP*	Valosin-containing protein	Ubiquitin-dependent protein degradation and autophagy	AD	Anterior lower leg	Rimmed vacuoles

## NOVEL GENES

### SMPX

Small muscle protein X-linked (*SMPX*), is predominantly expressed in skeletal muscle and heart, and localized in the costameric and intermyofibrillar region showing highest expression in slow muscle fibers [5]. Functionally, *SMPX* is thought to be part of the muscle cell regulatory network that coordinates the structural and functional states during growth, adaptation, and repair of these cells [5]. In particular, the protein protects the sarcolemma from mechanical stress [6]. In addition to the expression in muscle tissue, *SMPX* is expressed at low levels in various other organs, including various tissues of the inner ear. In 2011, the gene was first associated with human disease when protein truncating variants, and thus the loss of the protein in males, were identified in two unrelated families with X-linked deafness [7,8].

*SMPX* was first associated with skeletal muscle disease in 2021, making it one of the most recent additions to the list of genes known to cause distal myopathy. Through deep phenotyping and high-throughput sequencing, Johari and colleagues identified four different missense mutations in nine families from five different countries [9]. The molecular analysis suggested that two of these mutations were founder mutations: p.S78N likely originating from Malta, and p.P27A from France. The authors described a novel type of distal myopathy where the phenotype was highly consistent across the identified families. Clinically it was characterized by slowly progressive adult-onset distal muscle weakness where the ability to walk was preserved. The weakness typically started in the forearms with finger extension defect and in the lower legs with ankle dorsiflexion weakness and progressed to scapular arm abduction muscles while sparing the heart and respiratory muscles. Muscle imaging across several patients showed a pattern of muscle involvement where fatty replacement affected the anterior compartment muscles of the lower legs. Later, the calf muscles, medial gastrocnemius and soleus became affected, and then thigh muscle adductors and quads underwent fatty replacement more than the hamstrings. Histopathological analysis and electron microscopy of patient muscle biopsies showed rimmed vacuoles and sarcoplasmic inclusions-protein aggregates, some with amyloid-like characteristics. Recently, Salman *et al.*, also described an individual with the founder pathogenic variant p.S78N and prominent paravertebral muscle involvement. The diagnosis was facilitated by the patient's geographic origin, which served as an important clue for genotype–phenotype correlation [10].

### DNAJB2

*DNAJB2* encodes the J-domain cochaperones *DNAJB2a* and *DNAJB2b*. J-domain proteins (JDPs) are a group of cochaperones that play a critical role in protein quality control. Their J-domain stimulates the ATPase activity of heat shock protein A (HSPA) chaperones, which allows the chaperone cycle to bind and release substrate proteins [11,12]. *DNAJB2* has the highest expression in neurons, whereas lower expression has been shown in various other tissues [13]. DNAJB2b is the predominant isoform in most tissues; however, in skeletal muscle, the expression levels of the two isoforms is approximately equal [14]. Additionally, *DNAJB2* has some functions independent from the HSPA system, and for example, it has been shown to counteract TDP-43 aggregation [15]. Previously, variants in *DNAJB2* have been associated with variety of recessive progressive peripheral axonal neuropathies [16–23]. Recently, however, Liu *et al.*[24^▪^] described a rimmed-vacuolar myopathy in combination with distal hereditary motor neuropathy (dHMN) in a patient carrying homozygous missense variants in *DNAJB2*. Further, in 2023, the first dominantly inherited disease associated with *DNAJB2* was identified by Sarparanta and colleagues. They reported a patient with a combination of late-onset sensorimotor polyneuropathy and distal myopathy caused by a C-terminal frameshift variant, resulting in an extended protein [25^▪^]. Clinically, the proband presented with bilateral lower leg muscle weakness with both neurogenic and myopathic degenerative changes visible in muscle MRI. Muscle biopsy revealed fiber splitting, internalized myonuclei, and rimmed vacuoles, along with severe neurogenic changes.

### *RILPL1* and *ABCD3*

Oculopharyngodistal myopathy (OPDM) is a subtype of distal myopathy where weakness and atrophy of the distal limb muscles is combined with facial and bulbar muscle involvement [26]. So far, repeat expansion in five different genes have been identified as causative of OPDM: *GIPC1*, *LRP12*, *NOTCH2NLC*, and most recently, *RILPL1* and *ABCD3,* along with a noncoding CGG repeat expansion in LOC642361/NUTM2B-AS1 [27,28,29^▪▪^,30^▪^,^31^].

*RILPL1*, RAB-interacting lysosomal protein-like-1, is a centrosomal and ciliary protein that regulates lysosomal morphology [32,33]. It regulates protein localization in the primary cilium, including protein transport away from the cilia [32]. It can also inhibit ciliogenesis by binding RAB10 following LRRK2-mediated RAB10 phosphorylation [34]. In 2022, Yu *et al.*[29^▪▪^] identified a CGG repeat expansion in a previously unsolved family with OPDM. The authors achieved the identification of this CGG repeat in the 5′-UTR of *RILPL1* through a combination of long-read genome sequencing (LRS), repeat-primed PCR (RP-PCR), and fluorescence amplicon length analysis PCR (AL-PCR). This variant is associated with a novel OPDM phenotype (OPDM 4, MIM:619790), where distal limb muscle weakness develops more slowly than in other types of OPDM. Histopathological analysis of muscle tissue from OPDM 4 patients showed rimmed vacuoles, which is consistent with other types of OPDM.

Zheng *et al.*[35^▪▪^] identified additional six patients from two unrelated families with similar CGG repeat expansion in the same locus, resulting in OPDM 4. The authors noted an earlier disease onset in their patients compared with other types of OPDM. Across both studies, all patients were of Chinese origin, suggesting a founder effect; however, according to Zheng and colleagues, this has not been conclusively proven.

*ABCD3* is a ubiquitously expressed protein that belongs in the family of ATP-binding cassette proteins [36]. It is an ATP-dependent transporter with broad substrate specificity that catalyzes the transport of various fatty and bile acids from the cytosol to the peroxisome lumen for beta-oxidation [37]. Recently, Cortese *et al*[38^▪^]. described CCG repeat expansions in 5′-UTR of *ABCD3* in individuals across eight unrelated families of European ancestry, affected by OPDM. This was achieved through a combination of linkage studies, short-read genome sequencing, and targeted Oxford Nanopore-sequencing. This study marks the identification of the first repeat-expansion-related OPDM in individuals of non-Asian ancestry. European individuals with CCG ABCD3-related OPDM showed a similar phenotype compared with the Asian OPDM families reported earlier. These patients presented with late-onset distal myopathy where ptosis was usually the initial symptom. Histological findings from patient muscle biopsies showed rimmed vacuoles without intranuclear inclusions, which, again, aligned with previous findings in repeat-expansion-related OPDMs.

GCC-CGG repeats in several functionally unrelated genes have been identified as the genetic cause of OPDM. Thus, Cortese, Beecroft and colleagues suggest these OPDMs share an underlying pathogenic mechanism that is at least partly independent of the gene where the repeats are located. In a recent abstract, Li *et al.*[39] suggest that this disease mechanism may be related to repeat associated non-AUG initiated (RAN) translation. RAN translation-related disease mechanism has previously been described in relation to a 5′-UTR repeat expansion in *FMR1*, causing cerebellar ataxia [40].

### HSPB6

Sarparanta and colleagues recently identified a novel form of proximal and distal myopathy caused by a C-terminal protein extending frameshift variant in *HSPB6* (in preparation).

## MULTISYSTEM PROTEINOPATHIES

### HNRNPA1

Heterogeneous nuclear ribonucleoprotein A1 (*HNRNPA1*) belongs in a family of ubiquitously expressed heterogenous nuclear ribonucleoproteins (hnRNPs). These RNA-binding proteins associate with pre-RNAs in the nucleus and influence premRNA processing, as well as other aspects of mRNA metabolism and transport, and consequently play a role in alternative splicing [41,42]. *HNRNPA1* was originally associated with multisystem proteinopathy and amyotrophic lateral sclerosis (ALS). Kim *et al.*[43] identified missense mutations in the prion-like domain of *HNRNPA1* that resulted in multisystem proteinopathy, while different missense variants in the same domain were shown to cause ALS. In 2021, the first distal myopathies associated with *HNRNPA1* were independently described by Hackman *et al.*[44] and Beijer *et al.*[45]. Although the patients described by Beijer and colleagues presented with some distal myopathy characteristics, it is unclear whether their disease should be categorized under primary distal myopathies. Conversely, the family described by Hackman and colleagues presented with a clearer distal myopathy phenotype: the muscle weakness began in the small hand muscles, then spread to lower legs, and finally, to proximal muscles. The authors identified a small deletion in *HNRNPA1* in a previously unsolved Finnish family, originally reported by Mahjneh and colleagues (2003). The identification of this small, 160-base-pair deletion was achieved through a linkage analysis with single nucleotide polymorphism arrays and genome sequencing. Analysis of the patient muscle biopsy showed rimmed vacuoles and cytoplasmic inclusions at an advanced stage of the disease. RNA sequencing showed that the mutant allele produces a shorter mRNA compared with the wild-type allele.

In 2022, Chompoopong *et al.*[46] reported a novel heterozygous missense variant in *HNRNPA1* in a patient affected by bilateral foot drop without Paget disease or dementia. Muscle MRI showed abnormalities consistent with findings reported by Hackman and colleagues in their patients.

### TARDBP

TDP-43, encoded by *TARDBP*, is a ubiquitously expressed RNA-binding and DNA-binding protein. TDP-43 is involved in various steps of RNA biogenesis and processing, including the regulation of RNA splicing [47]. TDP-43 regulates the splicing of many protein-coding RNAs involved in neuronal survival and neurodegenerative diseases [48]. Interestingly, it has also been shown to participate in the skeletal muscle formation and regeneration by forming cytoplasmic myo-granules and binding mRNAs that encode sarcomeric proteins [49]. TDP-43 interacts with *HNRNPA1*, and this interaction is mediated by the C-terminal glycine-rich domain of the latter protein [43]. Similar to *HNRNPA1*, variants in *TARDP* have a long-standing association with familial ALS [50]. The vast majority of these causative variants are missense mutations in the C-terminal prion like domain (PrLD) [51–53]. In 2023, Zibold *et al.*[54^▪▪^] identified the first distal myopathy caused by a mutation in *TARDBP.* They identified a novel missense variant in PrLD in two French families affected by late-onset distal myopathy without ALS. Patient muscle biopsies revealed rimmed vacuoles, disruption of sarcomere integrity, and myofibrillar disorganization. Clinically, the presentation of the muscle weakness was often asymmetric with preferential initial involvement of the anterior compartment of the forearm. Within 5–10 years, the weakness spread to all four extremities, and eventually, in most patients, to bulbar and respiratory musculature. Most patients did not require nutritional or ventilatory support, and the ability to walk was usually preserved.

Another *TARDBP* mutation causing myopathy was recently identified by Ervilha Pereira *et al.*[55]. Using genome-wide linkage analysis and exome sequencing, they identified a C-terminal frameshift mutations producing an altered PrLD in a large family with rimmed vacuole myopathy. However, the authors describe a disease phenotype where both distal and proximal muscle were affected, and thus it is unclear if this disease should be categorized under distal myopathies.

## SAME GENE, DIFFERENT PATTERNS OF INHERITANCE

### ACTN2

*ACTN2* encodes alpha-actinin-2, a structural protein expressed in both cardiac and skeletal muscle sarcomeres [56]. The primary function of the protein is to cross-link actin in the sarcomere Z-disk. In addition, alpha-actinin-2 dynamically interacts with numerous other sarcomeric proteins, most notably with titin [57–59]. *ACTN2* has a long-standing association with cardiomyopathy, however, the first patients with *ACTN2*-related distal myopathies were first identified in 2019 by Savarese and colleagues [60], making the gene one of the more recent additions to the growing list of distal-myopathy-associated genes. Soon after, *ACTN2* was also associated with congenital myopathy [61]. Clinically, *ACTN2*-related diseases present with significant clinical heterogeneity, and clear genotype–phenotype correlations have not been established [62]. Generally, *ACTN2*-related distal myopathies begin with foot drop due to muscle weakness in the anterior compartment of the lower leg. Later, the weakness spreads to the thigh and posterior lower leg, leading to walking difficulties. However, this progression is usually slow, and upper limbs often remain unaffected [63]. Facial weakness has been reported in some distal myopathy patients with frameshift mutations causing a C-terminal protein extension [60,64,65]. In a manuscript published as a preprint, Ranta-Aho *et al.*[66] reported a large family with an *ACTN2* protein-extending variant presenting with a mixed phenotype where some patients were affected by skeletal myopathy, some by cardiomyopathy, and some by both.

Until recently, only autosomal dominant forms of *ACTN2*-related distal myopathies had been described. In 2021, Inoue *et al.*[67^▪▪^] identified the first recessive form of *ACTN2*-related distal myopathy. The authors describe eight patients from three unrelated families with a biallelic missense variant in *ACTN2.* These patients presented with fatty replacement in the anterior compartment of the lower leg. However, posterior compartment of the thigh and medial head of the gastrocnemius were also affected, whereas the anterior compartment of the thigh was largely spared. This pattern of muscle weakness somewhat deviates from the previously described dominant forms. Muscle biopsy showed scattered fibers with internal nuclei, rimmed vacuoles, nemaline bodies, and type 1 fiber predominance with minicore-like structures.

More recently, Donkervoort *et al.*[68^▪^] reported another recessive form of *ACTN2*-related distal myopathy, associated with a different missense variant. The authors describe seven patients from five families all manifesting with a consistent phenotype of distal lower extremity predominant muscle weakness. All patients were of Palestinian ancestry, and through haplotype analysis, the authors showed that this *ACTN2* variant is a founder mutation. A distinct pattern of asymmetric muscle weakness was observed: hamstrings and adductors in the thigh, and anterior compartment of the lower and soleus all were affected. Muscle biopsies showed disruption of the intermyofibrillar architecture, rimmed vacuoles, internal nuclei and type 1 fiber predominance.

## CONCLUSION

Distal myopathies are a highly heterogenous group of disease with variable age of onset, pattern of muscle weakness, and histological findings. During the past 10–15 years, more advanced sequencing methods have a contributed to the identification novel distal myopathy genes, which has only further emphasized this clinical heterogeneity. It has also brought on new diagnostic challenges: with vast amounts of data, efficient data analysis and variant interpretation has become increasingly complex. Some of the most recently identified distal myopathy genes elucidate this diagnostic challenge at hand: some of them lack an obvious functional connection to muscle tissue (e.g. *RILPL1*), have previously been associated with an unrelated clinical phenotype (e.g. *SMPX*) or harbor highly elusive variants (e.g. *HNRNPA1*), and thus, are extremely difficult to identify. Other known genes may present a challenge in finding genotype–phenotype correlation and variant interpretation, presenting with both autosomal dominant and recessive forms and significant clinical heterogeneity (e.g. *ACTN2*). The identification of novel distal myopathy genes and elusive variants is crucial in order to improve diagnostic rate in distal myopathies, and more research is needed to uncover these unknown causative genes.

## Acknowledgements

*None.*

### Financial support and sponsorship

*J.R.-A. is supported by Emil Aaltonen foundation. M.J. is supported by the Association Française contre les Myopathies (AFM Téléthon, The French Muscular Dystrophy Association, grant award number: 24438). B.U. is supported by Juselius foundation, Jane and Aatos Erkko foundation, and Samfundet Folkhälsan.*

### Conflicts of interest

*There are no conflicts of interest.*

## REFERENCES AND RECOMMENDED READING

Papers of particular interest, published within the annual period of review, have been highlighted as:

▪ of special interest
▪▪ of outstanding interest
